# Inhibition of the Cardiac Na^+^ Channel Nav1.5 by Carbon Monoxide*

**DOI:** 10.1074/jbc.M114.569996

**Published:** 2014-04-09

**Authors:** Jacobo Elies, Mark L. Dallas, John P. Boyle, Jason L. Scragg, Adrian Duke, Derek S. Steele, Chris Peers

**Affiliations:** From the ‡ Division of Cardiovascular and Diabetes Research, Leeds Institute of Genetics, Health and Therapeutics, Faculty of Medicine and Health and; § Institute of Membrane and Systems Biology, Faculty of Biological Sciences, University of Leeds, Leeds LS2 9JT, United Kingdom

**Keywords:** Carbon Monoxide, Heart, Nitric Oxide, Patch Clamp Electrophysiology, Sodium Channels, Arrhythmia

## Abstract

Sublethal carbon monoxide (CO) exposure is frequently associated with myocardial arrhythmias, and our recent studies have demonstrated that these may be attributable to modulation of cardiac Na^+^ channels, causing an increase in the late current and an inhibition of the peak current. Using a recombinant expression system, we demonstrate that CO inhibits peak human Nav1.5 current amplitude without activation of the late Na^+^ current observed in native tissue. Inhibition was associated with a hyperpolarizing shift in the steady-state inactivation properties of the channels and was unaffected by modification of channel gating induced by anemone toxin (rATX-II). Systematic pharmacological assessment indicated that no recognized CO-sensitive intracellular signaling pathways appeared to mediate CO inhibition of Nav1.5. Inhibition was, however, markedly suppressed by inhibition of NO formation, but NO donors did not mimic or occlude channel inhibition by CO, indicating that NO alone did not account for the actions of CO. Exposure of cells to DTT immediately before CO exposure also dramatically reduced the magnitude of current inhibition. Similarly, l-cysteine and *N*-ethylmaleimide significantly attenuated the inhibition caused by CO. In the presence of DTT and the NO inhibitor *N*^ω^-nitro-l-arginine methyl ester hydrochloride, the ability of CO to inhibit Nav1.5 was almost fully prevented. Our data indicate that inhibition of peak Na^+^ current (which can lead to Brugada syndrome-like arrhythmias) occurs via a mechanism distinct from induction of the late current, requires NO formation, and is dependent on channel redox state.

## Teaser

**Background:** CO poisoning causes cardiac arrhythmias, in part via modulation of the cardiac Na^+^ channel, Nav1.5.

**Results:** CO inhibition of peak recombinant Nav1.5 current occurs via nitric oxide formation and is also dependent on channel redox state.

**Conclusion:** CO inhibits peak recombinant Nav1.5 current via a mechanism distinct from activation of the late Na^+^ current.

**Significance:** CO may induce Brugada-like arrhythmias via inhibition of peak Na^+^ current.

## Introduction

Carbon monoxide (CO) is receiving much interest as an endogenous cell signaling molecule, and its role as a physiological regulator and potential therapeutic agent has been the subject of several recent reviews (*e.g.* Refs. 1–4). In the heart, both atrial and ventricular myocytes express the CO-generating enzymes HO-1 and HO-2 (HO-1 is induced by stress factors such as myocardial infarction (5)), and evidence suggests that some effects of endogenous CO may protect the myocardium, for example by limiting cellular damage caused by ischemia/reperfusion injury in the heart (6). Indeed, HO-1 knock-out increases (7) and HO-1 overexpression decreases (8) cardiac damage following ischemia/reperfusion injury. We have previously proposed that the cardioprotective effects of CO may in part arise due to its ability to reduce Ca^2+^ influx into myocytes via l-type Ca^2+^ channels (9).

Despite these advances in our understanding of the biology of CO, it remains an established environmental toxin, accounting for >50% of all fatal poisonings (10–12). The markedly different effects of exogenous and endogenous CO may reflect differences in the average tissue concentration or more subtle localized effects, present only when CO is produced intracellularly. Exogenous CO sources include motor exhaust fumes, gas appliances, wood burners, propane engines, and tobacco smoke. The myocardium is particularly susceptible to CO poisoning; chronic exposure to CO can induce myocardial injury and fibrosis (13–15), whereas acute exposure is associated with arrhythmias, which can in turn lead to sudden death (13, 16). CO also increases the likelihood of arrhythmias in patients with existing cardiac conditions (17, 18). Arrhythmic effects do not correlate with carboxyhemoglobin levels (13, 19), suggesting that tissue hypoxia does not account for its cardiotoxicity. Instead, electrocardiogram alterations in CO-exposed individuals indicate specific, pro-arrhythmic electrophysiological modifications (13, 20–22) which are mimicked in conscious rats (23). We recently proposed that CO-induced prolongation of the QT interval was attributable to induction of the late Na^+^ current, causing delay of repolarization. This arose due to CO-induced nitrosylation of the Na^+^ channel protein Nav1.5 following activation of endogenous NO formation (24). In that study, we also noted a reduction of the peak Na^+^ current, an effect that is also potentially arrhythmic because it is a feature of many forms of Brugada syndrome (25). To explore the mechanisms accounting for this important additional effect of CO on cardiac Na^+^ channels, we have examined the influence of CO on recombinant human Nav1.5. Our results indicate that CO reduces the peak Na^+^ current via a mechanism that is distinct from its action to induce the late Na^+^ current.

## EXPERIMENTAL PROCEDURES

### 

#### 

##### Cell Culture

HEK293 cells stably transfected with the full-length human cardiac sodium channel (SCN5A clone hH1, GenBank^TM^ accession no. M77235) were kindly provided by J. C. Makielski (University of Wisconsin) (26). Cells were cultured in growth medium comprising minimal essential medium with Earle's salts and l-glutamine, supplemented with 10% (v/v) fetal calf serum (Biosera, Ringmer, UK), 1% (v/v) nonessential amino acids, 1% (v/v) sodium pyruvate (Sigma), 50 μg/ml gentamicin, 100 units/ml penicillin G, 100 μg/ml streptomycin, and 0.25 μg/ml amphotericin in a humidified atmosphere of air/CO_2_ (19:1) at 37 °C. Selection pressure was maintained with G-418 (400 μg/ml). All culture reagents were purchased from Invitrogen unless otherwise stated.

##### nNOS Transfection

cDNA encoding rat neuronal nitric oxide synthase (rat nNOS;^2^ NM_052799) was originally excised from pcDNA3/rat nNOS (a kind gift from J. C. Makielski, University of Wisconsin) and subcloned (via 5′-EcoRI and 3′-XhoI) into pcDNA3.1(Neo^R^). To enable visualization of successfully transfected cells, rat nNOS (5′-NheI and 3′-XhoI) was then subsequently subcloned into pIRES-EGFP-puro (Addgene plasmid no. 45567; kindly deposited by Prof. Michael McVoy, Virginia Commonwealth University School of Medicine, Richmond, VA). HEK293 cells stably expressing Nav1.5 were transiently transfected with rat nNOS as follows: 1 × 10^6^ cells were resuspended in 100 μl of Nucleofector^R^ solution V (Lonza) at room temperature, 2 μg of DNA (pIRES-EGFP-puro/rat nNOS) were added, and the suspension was subjected to nucleofector^R^ program Q-01 as per the manufacturer's instructions (Lonza). The nNOS-transfected (GFP-positive) cells were sorted into sterile PBS containing 10% fetal calf serum on a Becton Dickenson FACSAria IIu using FACSDiva software (version 6.0). The suspension was then diluted in culture medium and transferred to coverslips at the appropriate density for electrophysiology or immunofluorescence analysis. Cells were used, or fixed, 24–48 h following transfection.

##### Immunofluorescence

Coverslips with transfected or untransfected cells were washed (2×) in Dulbecco's phosphate buffered saline (DPBS, Sigma-Aldrich) and then fixed with 4% paraformaldehyde in DPBS (20 min at room temperature). Coverslips were then washed (3× for 5 min) in DPBS. Cells were permeabilized by incubation with 0.05% Triton X-100 in DPBS containing 10% normal goat serum (for 20 min at room temperature). After washing, cells were incubated overnight at 4 °C with anti-nNOS (clone 3G6B10, Invitrogen, 1:250) in DPBS containing 1% normal goat serum. Cells were washed (3× for 5 min) and then incubated with Alexa Fluor^R^ 488 or 633 conjugated donkey anti-mouse (Invitrogen, 1:1000) in DPBS containing 1% normal goat serum. Coverslips were washed (3× for 5 min), mounted using Vectashield^R^ containing DAPI (Vector Laboratories, Burlingame, CA), and sealed with nail polish. Slides were examined on an LSM510 confocal microscope running Zen software (Carl Zeiss).

##### Electrophysiology

Whole-cell patch clamp recordings were used to study macroscopic Na^+^ currents at room temperature (23 ± 2 °C). Patch pipettes (2 to 5 megohms) were filled with the following: 120 mm CsF, 20 mm CsCl, 5 mm EGTA, buffered with 5 mm HEPES; pH was adjusted to 7.2 with CsOH. Cells attached to coverslips were placed in a perfused (2–4 ml/min) chamber using extracellular solution containing the following: 10 mm NaCl, 130 mm choline chloride, 4 mm KCl, 1.8 mm CaCl_2_, 0.75 mm MgCl, 10 mm glucose, buffered with 5 mm HEPES. Na^+^ levels were reduced to 10 mm to reduce the current amplitudes to levels that allowed accurate voltage clamp. Extracellular pH was adjusted to 7.4 with NaOH and osmolarity of both solutions was 300 mOsm. After gigaohm seal formation and establishment of whole-cell voltage clamp, whole-cell currents were recorded, digitized, and stored with an Axopatch 200B amplifier, Digidata 1322A, and pCLAMP 9 (Molecular Devices, Union City, CA). Currents were sampled at 50 kHz and low pass-filtered at 20 kHz. Series resistance was compensated by 70–90%.

Peak Na^+^ currents were recorded by stepping from a holding potential of −100 to −30 mV for between 40 ms and 400 ms. Start-to-start time between sweeps was 4 or 10s. *I-V* relationships were measured by stepping from a holding potential of −100 mV to voltages between −120 mV and +40 mV in 10-mV increments for 50-ms each (1 Hz). Time series studies employed repeated 50-ms step depolarizations ranging from −100 mV to −30 mV (0.1 Hz). Steady-state inactivation of Na^+^ currents was measured by applying pre-pulses ranging from −120 to −50 mV in 10-mV increments for 5 s prior to the test potential (−30 mV for 100 ms). Currents were measured at their peak and at 100 ms (for late current) following leak subtraction by the P/4 method. Analysis was performed using Clampfit 9 (Molecular Devices, Sunnyvale, CA), and subsequent curve-fitting and statistical analysis was undertaken using GraphPad Prism (version 4, GraphPad Software, La Jolla, CA).

##### Confocal DAF-2 Imaging

Cells were incubated for 60 min at 37 °C in extracellular solution composed of the following: 140 mm NaCl, 5 mm KCl, 1.5 mm CaCl_2_, 2 mm MgCl_2_, 10 mm HEPES, 11 mm glucose, pH 7.4) containing DAF-2 diacetate (5 μm), a cell membrane-permeable NO-sensitive fluorescent dye (27). Cells were then gently washed twice with extracellular solution and left for at least 15 min in an incubator to allow the hydrolysis of DAF-2 diacetate into the free NO-sensitive free acid form (DAF-2). Cells were placed on a Zeiss (Oberkochen, Germany) laser scanning confocal microscope (LSM 510) fitted with ×40 oil immersion lens (Zeiss Plan Neofluar, refractive index of 1.3) and continuously perfused at 0.5 ml/min. DAF-2 loaded cells were excited with the 488-nm line of a 20-mW diode laser (attenuated by ∼90%), and emitted fluorescence was measured at >515 nm. x-y images were obtained from isolated cells at 1-min intervals to minimize photobleaching using Zeiss AIM software. Fluorescence intensity was analyzed from isolated cells using ImageJ software. Identical settings were used for each test condition.

##### Drugs and Solutions

The NO donor (*S*)-nitroso-*N*-acetylpenicillamine and SB203580 were purchased from Ascent Scientific (AbCam Chemicals, Cambridge, UK). SIN-1 hydrochloride, Mn(III)terakis(1-methyl-4-pyridyl) porphyrin tetratosylate hydroxide (MnTMPyP), and the NO donor *S*-nitrosoglutathione were purchased from Calbiochem (Merck Chemicals, Nottingham, UK). MitoQ was a gift from Dr. M. Murphy (Cambridge, UK). Allopurinol, antimycin A, l-ascorbic acid, choline chloride, DTT, diphenyleneiodonium chloride, 2,2′-dithiobis(5-nitropyridine), *N*-ethylmaleimide, the calcium- and calmodulin-dependent protein kinase II (CaMKII) inhibitor KN-93, reduced glutathione, *N*^ω^-nitro-l-arginine methyl ester hydrochloride (l-NAME), rotenone, stigmatellin, and superoxide dismutase were purchased from Sigma-Aldrich. Ebselen was purchased from Cayman Chemical (Ann Arbor, MI), and DAF-2 diacetate was purchased from Invitrogen. Anemone toxin ATX-II was purchased from Alomone Labs (Jerusalem, Israel). The PKG inhibitor Rp-8-Br-PET-cGMPS and PKG activator Sp-8-Br-PET-cGMPS were purchased from BIOLOG Life Science Institute (Bremen, Germany). DTT was added to the extracellular solution as a reducing agent, and *N*-ethylmaleimide was added to the intracellular solution as an alkylating agent. Where necessary, appropriate precautionary measures were taken throughout the experimental procedures to avoid degradation of light-sensitive compounds (ascorbic acid, trolox (Sigma), SIN-1, and MnTMPyP), as well as to avoid extensive photobleaching of fluorescent dye. Solution pH was measured before the start of each experiment because fluorescence of DAF derivatives is highly pH-dependent (28).

Pooled data are presented as mean ± S.E. Statistical comparisons were effected by using Student's *t* test and analysis of variance (where appropriate), with a value of *p* < 0.05 considered significant.

## RESULTS

### 

#### 

##### CO Inhibits the Peak Recombinant Nav1.5 Current

**FIGURE 1.**
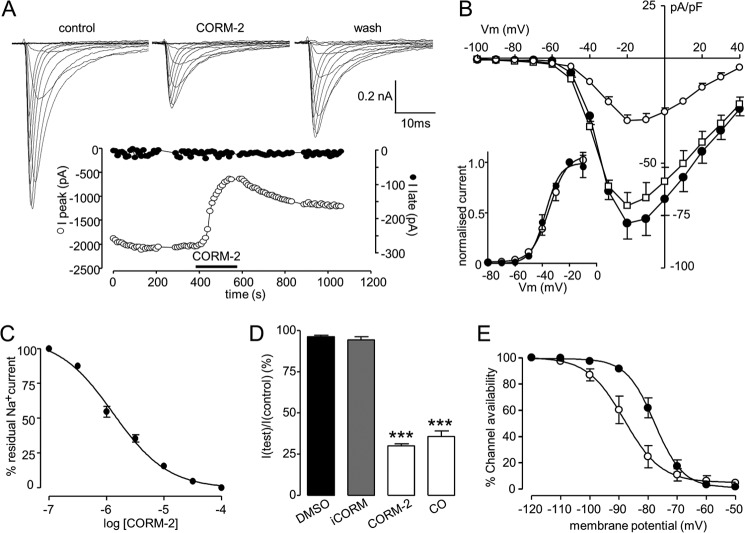
**CO inhibits recombinant human Nav1.5 channels expressed in HEK293 cells.** *A*, shown are families of currents evoked by step depolarizations applied from a holding potential of −100 mV to between −70 mV and +40 mV before (control) during (CORM-2) and after a 3-min washout of the CO donor CORM-2 (3 μm). Shown *below* is a time series plot in which each open plotted point is the measured peak amplitude of current evoked by successive step depolarizations from −100 mV to −30 mV. For the period indicated by the *horizontal bar*, the cell was exposed to 3 μm CORM-2. The *solid symbols* show the amplitude of the late current measured in the same cell. Gaps in the time series represent brief periods when current voltage relationships were recorded. *B*, mean ± S.E. (*error bars*) current density *versus* voltage plots determined in control cells (●; *n* = 7), cells exposed to 3 μm CORM-2 (○; *n* = 7), and cells exposed to 3 μm iCORM (□; *n* = 7). Effects of CORM were statistically significant (*p* < 0.02 to *p* < 0.0001) over the voltage range −40 to +40 mV. *Inset* plots show normalized current activation curves under control conditions (●; *n* = 7) and in the presence of 3 μm CORM-2 (○; *n* = 7). *C*, concentration response relationship indicating the potency of CORM-2 to inhibit Nav1.5 currents. Each point plotted is the mean ± S.E. (*error bars*) inhibition of current determined from seven cells in each case. The fitted curve revealed an IC_50_ value of 1.38 μm. *D*, bar graph showing mean ± S.E. (*error bars*) (*n* = 9 cells in each case) inhibition caused by solvent (dimethyl sulfoxide (*DMSO*); 0.1%), iCORM, CORM-2, and CO dissolved directly into solution. ***, *p* < 0.0001 *versus* control. *E*, steady-state inactivation curves determined in four cells before and during exposure to 3 μm CORM-2. Each point represents mean ± S.E. (*error bars*), and curves were fitted by Boltzmann equations. Effects of CORM were statistically significant (*p* < 0.01, *p* < 0.001) over the voltage range of −100 to −80 mV.

**FIGURE 2.**
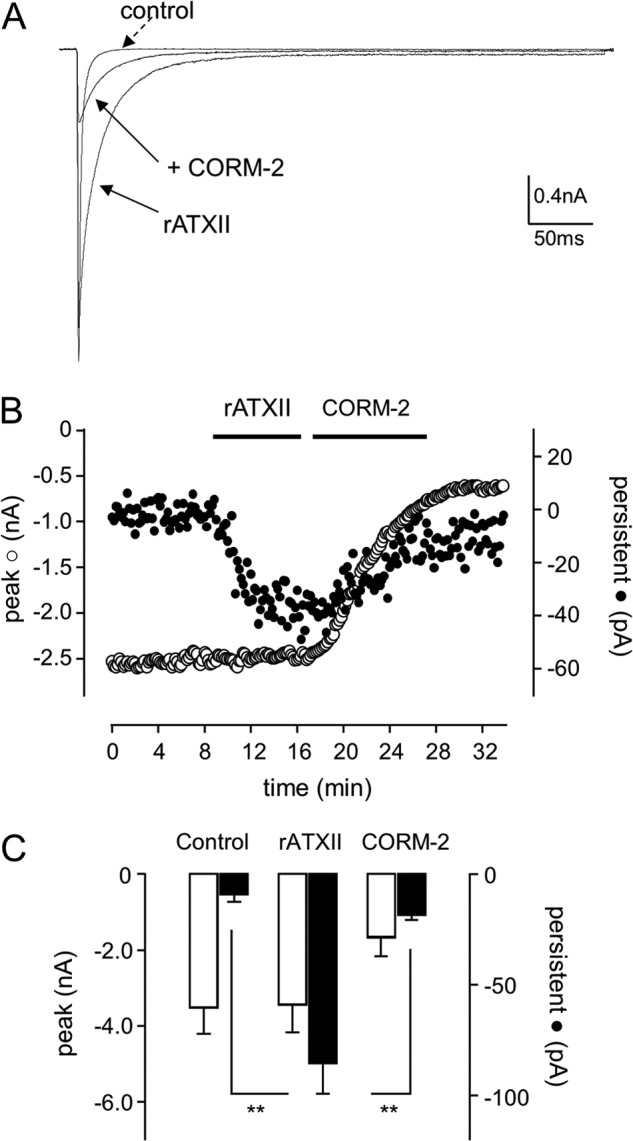
**CO inhibits the ATX-II induced late Nav1.5 current.** *A*, shown are currents evoked in a Nav1.5-expressing HEK293 cell under control conditions, during exposure to 50 nm rATXII, and during exposure to 3 μm CORM-2 following rATXII exposure, as indicated. Currents were evoked by step depolarizations from −100 to −30 mV. *B*, shown is a time series plot in which each open plotted point is the peak amplitude of current evoked by successive step depolarizations from −100 to −30 mV (measured 200 ms into pulse duration). For the periods indicated by the *horizontal bar*, the cell was exposed to 50 nm rATXII and then to 3 μm CORM-2. The *solid symbols* show the amplitude of the late current measured in the same cell. *C*, bar graph plotting mean ± S.E. (*n* = 5 cells) peak (*open bars*) and late (*solid bars*) current amplitudes under control conditions and in the presence of 50 nm rATXII and then 3 μm CORM-2, as indicated. **, *p* < 0.01.

As illustrated in Fig. 1*A*, the CO donor CORM-2 caused marked inhibition of the peak Na^+^ current recorded in Nav1.5-transfected cells. Effects were slow to reverse, with only 34.1 ± 5.0% recovery seen after 3-min washout (*n* = 10 cells). Strikingly, potentiation of the late current was not apparent (Fig. 1*A*; detailed further in Fig. 2). Current inhibition was observed at all activating test potentials (Fig. 1*B*), and there was no significant effect on the voltage dependence of activation (Fig. 1*B*, *inset*), with an IC_50_ of 1.38 μm CORM-2 (Fig. 1*C*). The CORM-2 solvent, dimethyl sulfoxide (0.1%) was without significant effect on Na^+^ currents, as was the inactive compound, iCORM (Fig. 1, *B* and *D*). CO dissolved directly into solution as described previously (24) to a concentration of 87 μm, was as effective as CORM-2 in inhibiting currents (Fig. 1*D*). Associated with the inhibition of currents by CORM-2 (3 μm) was a hyperpolarizing shift of the steady state inactivation profile of the current, from a *V*_0.5_ of −76.0 ± 2.2 mV to −89.5 ± 0.81 mV (Fig. 1*E*, *n* = 4; *p* < 0.001).

The lack of effect of CO on the late Na^+^ current is in striking contrast to its induction by CO in native tissue (24), suggesting that the inhibition of the peak Na^+^ current occurs via a mechanism distinct from late Na^+^ current induction. It is conceivable that heterologous expression of the Nav1.5 α subunit, in the absence of auxiliary native proteins, may be unable to generate a late current. To investigate this, we examined the response of the recombinant Nav1.5 to anemone toxin (ATX-II), which can induce the late current in native tissue (29). As shown in Fig. 2, *A–C*, bath application of 100 nm rATX-II caused an induction of the late (persistent) Na^+^ current, without affecting the amplitude of the peak current. Effects of the toxin were essentially irreversible (data not shown) as reported previously (30). However, subsequent exposure to 3 μm CORM-2 significantly reduced both peak and persistent Na^+^ current amplitude (Fig. 2, *A–C*), suggesting it inhibits Nav1.5 regardless of the gating alterations caused by rATX-II.

##### Probing the Mechanism of CO Inhibition of Nav1.5

**FIGURE 3.**
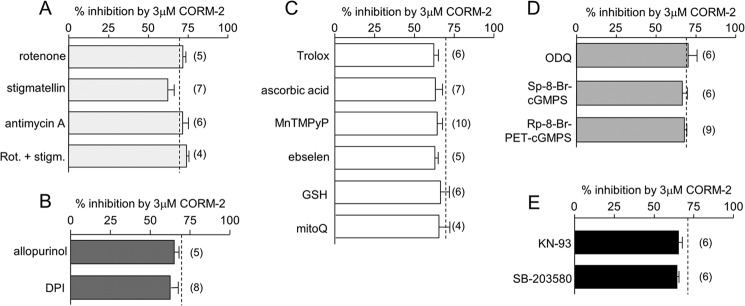
**CO inhibits Nav1.5 without involvement of numerous known signaling pathways.** Bar graphs show the effects of various agents that interfere with the ability of CO (applied as CORM-2, 3 μm) to reduce the peak Na^+^ current amplitude. In each case, mean effects of CORM-2 were determined from the number of cells (indicated in *parentheses*). *Dashed lines* indicate the mean effects of CORM-2 applied alone (*i.e.* in the absence of other agents; 71.1 ± 0.48% inhibition, *n* = 15)). Agents investigated were designed to probe the involvement of mitochondria. *A*, 2 μm rotenone (*Rot.*), 1 μm stigmatellin (*stigm.*; 30 min preincubation), and 3 μm antimycin A. *B*, xanthine oxidase and NADPH oxidase (1 μm allopurinol (30-min preincubation), and 3 μm diphenyleneiodonium chloride (*DPI*)); *C*, antioxidants (400 μm trolox; 500 μm ascorbic acid; 100 μm MnTMPyP, and 1 μm ebselen (30-min pre-incubation), 3 mm glutathione (*GSH*), and 250 nm mitoQ); *D*, cGMP/PKG pathway (30 μm 1*H*-[1,2,4]oxadiazolo[4,3-*a*]quinoxalin-1-one (*ODQ*), 0.5 μm Sp-8-Br-cGMPS, and 0.5 μm Rp-8-Br-PET-cGMPS (1-h preincubation for these three drugs)); *E*, involvement of other kinases (1 μm KN-93, and 10 μm SB-203580 (1-h preincubation for both drugs)).

CO exerts a range of effects in different tissues via numerous diverse signaling pathways (1–4). We systematically explored the possible involvement of these pathways using a variety of pharmacological interventions, and results are summarized in Fig. 3. Mitochondria are a major intracellular source of ROS via electron “leak” from the electron transport chain (31), and CO appears to increase ROS production by interacting with cytochrome *c* oxidase (9, 32–34). For these reasons, and because ROS levels have been reported to modulate cardiac Na^+^ channel activity (35), we examined the effects of CO in the presence of known electron transport chain inhibitors. As shown in Fig. 3*A*, an inhibitor of complex I (rotenone, 2 μm), two inhibitors of complex III (stigmatellin (1 μm, 30 min of preincubation) and antimycin A (3 μm)), and the combined presence of rotenone and stigmatellin were unable to modulate the response to CO. Similarly, the ability of CO to inhibit the peak Na^+^ current was unaffected by allopurinol (1 μm, 30 min of preincubation) and diphenylene iodonium (3 μm), discounting the involvement of ROS derived from xanthine oxidase or NADPH oxidase (Fig. 3*B*).

We then tested a range of general antioxidants: trolox (400 μm), ascorbic acid (500 μm), MnTMPyP (100 μm), ebselen (1 μm, 30 min of preincubation), reduced glutathione (3 mm), and the mitochondria-targeted antioxidant, mitoQ (250 nm, 30 min of preincubation). None of these were effective in altering the ability of CORM-2 to inhibit currents (Fig. 3*C*).

CO has been shown to activate the cGMP/PKG pathway to exert some of its effects (36). We therefore examined its ability to inhibit Nav1.5 currents in the presence of the soluble guanylate cyclase inhibitor 1*H*-[1,2,4]oxadiazolo[4,3-*a*]quinoxalin-1-one (ODQ; 30 μm, 1 h of preincubation), and a membrane-permeant cGMP activator (Sp-8-Br-cGMPS; 0.5 μm, 1 h of preincubation) and inhibitor Rp-8-Br-PET-cGMPS (0.5 μm, 1 h of preincubation). Each compound was ineffective in altering the ability of CORM-2 to inhibit Nav1.5 (Fig. 3*D*). Similarly, inhibition of Nav1.5 currents by CORM-2 was unaltered in the presence of the CaMK II inhibitor KN-93 (1 μm) or the p38 MAPK inhibitor SB-203580 (10 μm, 1 h of preincubation). These experiments suggest that these known CO-sensitive targets are not involved in the inhibition of Nav1.5 (Fig. 3*E*).

##### CO Inhibition of Nav1.5 Requires NO Formation

**FIGURE 4.**
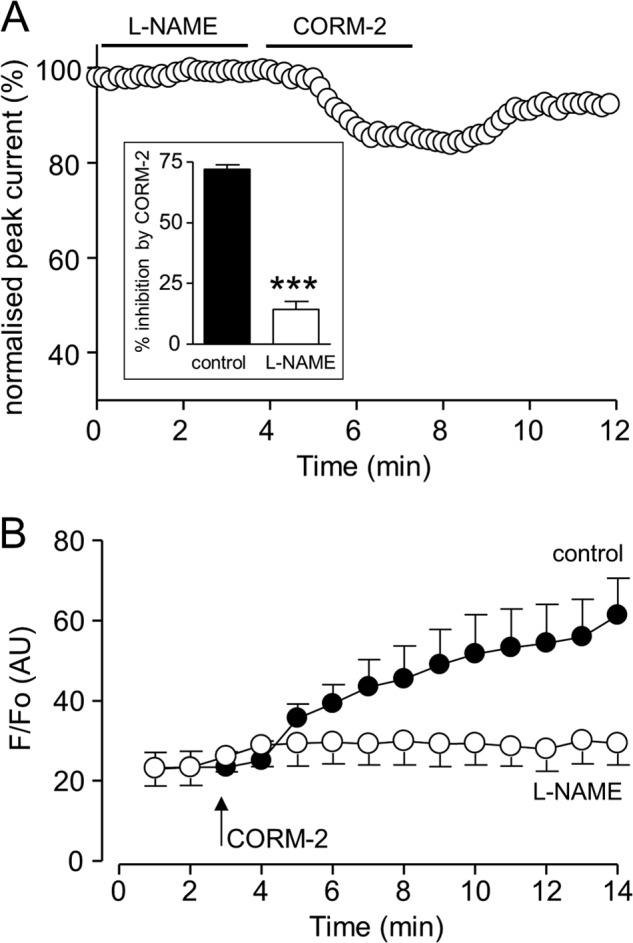
**CO inhibition of Nav1.5 involves NO formation.** *A*, shown is a time series plot in which each plotted point is the peak amplitude of current evoked by successive step depolarizations from −100 to −30 mV. For the periods indicated by the *horizontal bars*, the cell was exposed to 1 mm l-NAME and then to 3 μm CORM-2. *Inset*, bar graph showing the mean ± S.E. effects of 3 μm CORM-2 alone (*n* = 10), or following pretreatment with l-NAME (*n* = 10). ***, *p* < 0.0001. *B*, DAF-2 fluorescence measured in HEK293 cells overexpressing Nav1.5. At the point indicated by the *arrow*, cells were exposed to 3 μm CORM-2. Mean ± S.E. fluorescence (plotted as arbitrary units, *AU*) is shown for naive cells (*solid circles*) or cells pretreated with 1 mm l-NAME (*open circles*; *n* = 5 in each case). Fluorescence was statistically significantly different between the two groups at *t* = 9 min and later (*p* < 0.05 to *p* < 0.001).

**FIGURE 5.**
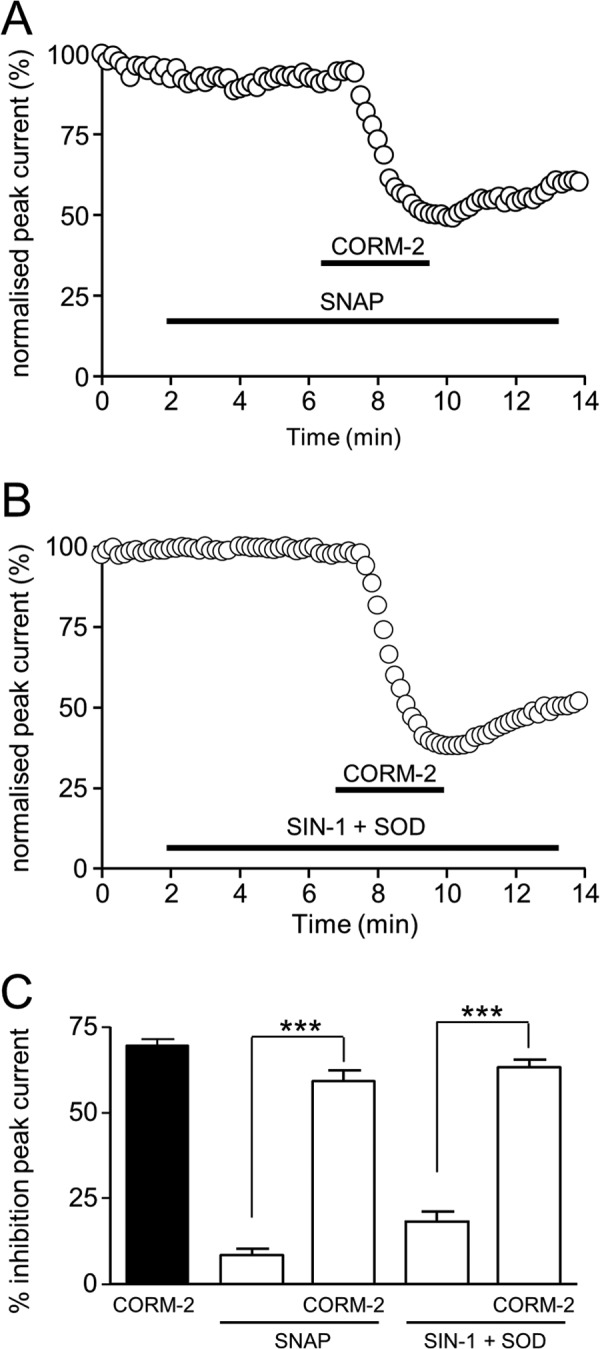
**NO donors do not mimic CO.** *A*, shown is a time series plot showing the measured peak current amplitude evoked by successive step depolarizations from −100 to −30 mV. For the periods indicated by the *horizontal bars*, the cell was exposed to 200 μm (*S*)-nitroso-*N*-acetylpenicillamine and to 3 μm CORM-2, as indicated. *B*, as in *A*, except that the cell was exposed to 100 μm SIN-1 plus superoxide dismutase (*SOD*; 50 units/ml) rather than (*S*)-nitroso-*N*-acetylpenicillamine (*SNAP*). *C*, bar graph showing mean ± S.E. inhibition of peak Na^+^ current (measured as in *A* and *B*) caused by 3 μm CORM-2 applied alone (*solid bar*) or together with the NO donors as used in *A* and *B*, and the effects of the donors alone (*n* = 5 cells in each case). ***, *p* < 0.0001.

We extended the investigation to examine a possible involvement of NO because CO is an established activator of NO production, possibly by activating NOS (2, 37). In the presence of l-NAME, which prevents NO formation, the ability of CO to inhibit Nav1.5 was significantly reduced, although not abolished (Fig. 4, *A* and *B*). This finding prompted us to investigate whether CO can stimulate NO formation, as is the case in cardiac myocytes (24). To this end, we loaded cells with the NO-sensitive fluoroprobe DAF-2 and found that exposure to CORM-2 produced a time-dependent increase in the production of NO, which was prevented by l-NAME (Fig. 4*B*). Thus, CO inhibition of Nav1.5 is in part dependent on its ability to induce NO formation. However, despite the dependence of the effects of CO on NO formation, application of NO donors was not sufficient to mimic the actions of CO. As exemplified in Fig. 5, *A* and *B*, application of (*S*)-nitroso-*N*-acetylpenicillamine (200 μm; Fig. 5*A*) or SIN-1 (100 μm, applied together with superoxide dismutase (50 units/ml) to prevent peroxynitrite formation; Fig. 5*B*) only exerted minimal effects on peak Nav1.5 current amplitude, and their presence did not significantly alter the ability of CO to inhibit currents (Fig. 5*C*).

**FIGURE 6.**
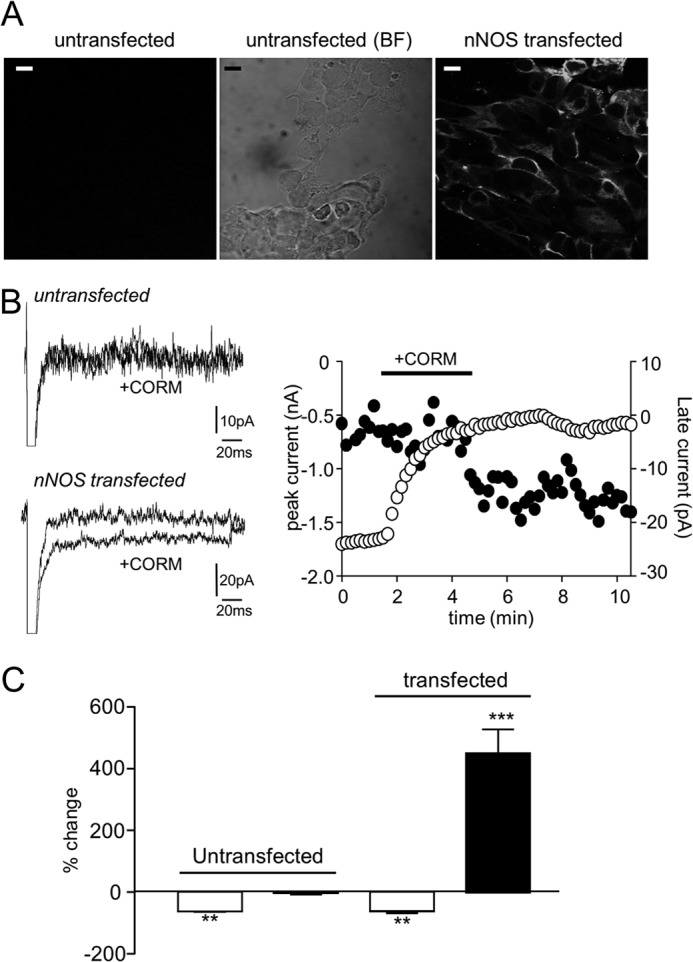
**Co-expression of nNOS permits CO-induction of the late Na^+^ current.** *A*, immunofluorescent (*left*) and bright field (*BF*; *center*) images of Nav1.5-expressing HEK293 cells without co-expression of nNOS. *Right image* shows immunofluorescence staining for nNOS in cells transiently transfected with nNOS and eGFP on the pIRES-EGFP-puro plasmid. *Scale bars* represent 10 μm in each case. *B*, *left*, shown are currents evoked in a non-transfected cell (*upper traces*) and a nNOS-transfected cell (*lower traces*) before and during exposure to 3 μm CORM-2, as indicated. Currents evoked in each case by step depolarizations from −100 to −30 mV. *Right*, shown is a time series plot showing the measured peak (*open symbols*) and late (*solid symbols*) current amplitudes evoked by successive step depolarizations from −100 to −30 mV. For the period indicated by the *horizontal bar*, the cell was exposed to 3 μm CORM-2, as indicated. The cell underwent transfection with nNOS and demonstrates the late current enhancement by CO exposure only seen in these transfected cells. *C*, bar graph showing mean ± S.E. effects of 3 μm CORM-2 on peak Na^+^ current (*open bars*) and late current (*solid bars*) caused by applied to untransfected cells (*n* = 5) and nNOS-transfected cells (*n* = 6). **, *p* < 0.01; ***, *p* < 0.0001.

Ueda *et al.* (38) have demonstrated that in cardiac myocytes native Na^+^ channels form part of a multimeric protein complex that includes the neuronal form of nitric oxide synthase, nNOS. Furthermore, NO derived from nNOS increases the late Na^+^ current in HEK293 cells co-expressing Nav1.5 α (but not auxiliary β) subunits and nNOS (38). To investigate a possible involvement of nNOS in mediating the effects of CO on Nav1.5-mediated currents in our HEK293 cells, we co-expressed nNOS using an eGFP-coexpressing plasmid. As shown in Fig. 6*A* (*left*), nNOS was not detected in untransfected HEK293 cells visually by immunocytochemistry. However, all transiently transfected cells, following FACS analysis, consistently expressed nNOS (and eGFP; data not shown). This was consistently observed in each of four transfections (*e.g.* Fig. 6*A*, *right*).

As described in Fig. 1, no late current was induced in non-transfected cells (Fig. 6*B*, *upper traces*, Fig. 6*C*). By contrast, in all six cells studied following transfection with nNOS, exposure to CO (3 μm) inhibited peak Na^+^ current by 60.4 ± 2.4% (comparable with the effects seen in untransfected cells; Fig. 1) but also caused a striking increase in the late current amplitude (Fig. 6*B*, *left*, *lower traces*, and accompanying time series, Fig. 6*B*, *right*). Mean data are shown in Fig. 6*C*. These data are consistent with the idea that CO can induce the late Na^+^ current in HEK293 cells only when they co-express nNOS but that nNOS expression is not required for peak current inhibition. Thus, they further support the notion that CO inhibition of the peak Na^+^ current is mechanistically distinct from its ability to modulate the late Na^+^ current and requires NO from a distinct source.

To investigate possible effects on inactivation kinetics, we fitted the decay phase of nNOS-transfected cells with a double exponential function before and during exposure to CO. The fast time constant was slightly but significantly (*p* < 0.05) slowed from 1.47 ± 0.12 ms to 1.99 ± 0.17 ms in the presence of 3 μm CORM-2 (*n* = 6), whereas the slow time constant was unaffected (8.61 ± 1.37 ms *versus* 9.39 ± 1.75 ms).

##### Nav1.5 Redox Status Influences Sensitivity to CO Modulation

**FIGURE 7.**
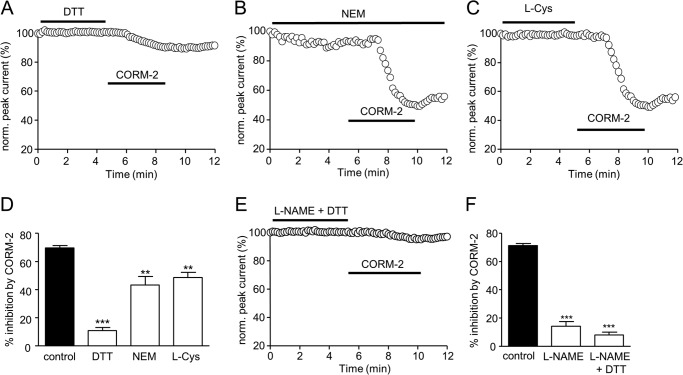
**Cysteine-modulating agents influence Nav1.5 inhibition by CO.** *A*, shown is a time series plot showing the measured peak current amplitude evoked by successive step depolarizations from −100 to −30 mV. For the periods indicated by the *horizontal bars*, the cell was exposed to 1 mm DTT and then to 3 μm CORM-2, as indicated. *B*, as described in *A*, except that the cell was dialyzed with 300 μm *N*-ethylmaleimide (*NEM*) rather than exposed to DTT. *C*, as described in *A* except that the cell was exposed to 100 μm l-cysteine (*L-Cys*) rather than DTT. *D*, bar graph showing mean ± S.E. inhibition of peak Na^+^ current (measured as in *A–C*) caused by 3 μm CORM-2 applied alone (*solid bar*) or together with the cysteine modifying agents used in *A–C*, and the effects of the donors alone (*n* = 10 for DTT, *n* = 9 for *N*-ethylmaleimide, and *n* = 8 for l-Cys). ***, *p* < 0.0001; **, *p* < 0.005. *E*, time series plot as in *A–C*, except that the cells were exposed to 1 mm l-NAME together with 1 mm DTT before exposure to 3 μm CORM-2. *F*, bar graph showing mean ± S.E. inhibition of peak Na^+^ current (measured as in *A–C*) caused by 3 μm CORM-2 applied alone (*solid bar*) of after treatment with 1 mm l-NAME alone (from Fig. 4) or together with DTT (*n* = 10 cells). ***, *p* < 0.0001.

Despite the lack of effect of antioxidants to interfere with the ability of CO to modulate Nav1.5 (Fig. 3), the channel is known to undergo regulation by more stringent reducing and oxidizing agents (39–41), suggesting reactive cysteine residues can regulate channel function. To examine whether they may influence channel sensitivity to CO, we first pretreated cells with such agents and then examined the subsequent response to CO exposure. As illustrated in Fig. 7*A*, application of 1 mm DTT (which alone had no significant effect on currents), dramatically reduced the subsequent ability of CORM-2 to inhibit peak Na^+^ currents (quantified in Fig. 7*D*). Similarly, intracellular dialysis with the cysteine alkylating agent *N*-ethylmaleimide (300 μm; which itself reduced currents by 8.0 ± 1.7%, *n* = 5) also significantly reduced the sensitivity to CORM-2 (Fig. 7, *B* and *D*), as did bath application of l-cysteine (Fig. 7, *C* and *D*). Following exposure to both l-NAME and DTT, CORM-2 was almost completely without effect on Nav1.5 (Fig. 7, *E* and *F*). Thus, our results indicate that CO inhibition of Nav1.5 is in part dependent on NO formation and is also dependent on the redox status of available channel cysteine residues.

## DISCUSSION

CO is being championed as a novel therapeutic approach in the treatment of numerous disorders, including cardiovascular disease, cancer, inflammation, and also to improve rejection rates following organ transplantation (42). Furthermore, clinical trials are currently determining its safety and usefulness in treating a variety of disorders (for more details, search clinicaltrials.gov for “carbon monoxide”) in humans. Despite these positive prospects, however, CO remains the most common form of death by poisoning (10, 12). The heart and brain (particularly in the aging population) are most susceptible to CO-induced damage (13, 43–45), and symptoms are difficult to diagnose (46). Experimentally, both positive and negative effects of CO can be demonstrated. Thus, CO can reduce neuronal damage arising from focal ischemia (47) yet can be detrimental through disruption of neuronal Ca^2+^ homeostasis (48). In the heart, endogenous CO arising from elevated HO-1 expression limits the cellular damage caused by ischemia/reperfusion injury in mice (6–8), and similar protective results have been recently reported using the CO donor, CORM-3 (24). By contrast, we have recently demonstrated that exogenous CO (applied directly or via CO donors CORM-2 or CORM-3) can have proarrhythmic actions at similar concentrations (24). These findings suggest that there may be a narrow therapeutic window for CO, at least as a treatment for the myocardium. It is imperative, therefore, that a full understanding of the molecular and cellular effects of CO is established before its usefulness as a therapeutic agent can be exploited.

We demonstrated recently that CO augmented the late Na^+^ current via increased NO production and subsequent nitrosylation of Nav1.5 channel protein (24). This effect led to early afterdepolarization-like Ca^2+^ transients in single myocytes and proarrhythmic effects *in vivo* that could precipitate ventricular fibrillation and death in the presence of isoprenaline (24). Although it was also noted, no exploration of the mechanism underlying the additional effect of CO to inhibit peak Na^+^ currents was provided. We did, however, report a hyperpolarizing shift in the steady-state inactivation curve for the native channel (24), as shown in Fig. 1*E* for the recombinant channel. Note, however, that the standard holding potential for most experiments was −100 mV, and at this potential changes in steady-state inactivation properties caused by CO can only account for ∼10% of the CO-mediated inhibition. This inhibition of peak Na^+^ current is clinically important because reductions of peak Na^+^ current amplitude (arising primarily from mutations in Nav1.5) account for a large fraction of arrhythmias seen in Brugada syndrome patients, regardless of whether amplitude reduction arises from disrupted channel trafficking or function (25, 49).

Through studying the effects of CO on recombinant Nav1.5 expressed in HEK293 cells, we can clearly distinguish the ability of CO to inhibit the peak Na^+^ current from its ability to increase the late component of the native Na^+^ current. Thus, CO retained the ability to inhibit the peak current, yet the late current was unaffected by CO (Figs. 1 and 2). This lack of effect on the late current was not due to an inability of the channel to display such activity because rATX-II caused a dramatic gating shift and the appearance of a late current. However, this was inhibited along with the peak current by CO (Fig. 2). Although the lack of late current induction by CO distinguishes its effects on the late from the peak current, it is noteworthy that both late current induction (24) and inhibition of peak current were largely mediated by formation of NO. Thus, in cardiomyocytes (24), HEK293 cells (Fig. 4), and other cell types (48), CO increased intracellular NO levels, and in each case, the effects of CO were prevented or dramatically reduced by inhibiting NO formation. However, it is equally noteworthy that although the augmentation of the late current by CO could be mimicked by an NO donor (24), the inhibition of the peak current could not (Fig. 5). This observation further distinguishes the mechanisms underlying the effects of CO on peak and late cardiac Na^+^ currents and agrees with an earlier report of a lack of effect of NO donors on expressed cardiac Na^+^ channels (50).

A previous study has indicated that cardiac Na^+^ channels exist in a protein complex that also includes nNOS and that constitutive activity of nNOS (arising from a mutation of the scaffolding protein syntrophin, also present in the complex) leads to channel nitrosylation and induction of the late Na^+^ current (38). When we co-expressed cells with nNOS (Fig. 6), CO was indeed able to increase the late Na^+^ current. This experiment demonstrated that recombinant Nav1.5 channel α subunits could generate a late Na^+^ current in the absence of auxiliary subunits and that this effect was dependent on nNOS co-expression. Whether this was due simply to increased NO production or through specific colocalization of nNOS with Nav1.5, we do not know. However, most importantly, it further supported our conclusion that inhibition of the peak Na^+^ current, despite also being NO-dependent, occurred via a different pathway/mechanism to the induction of the late Na^+^ current.

Systematic pharmacological analysis discounted the involvement of ROS (from mitochondria and other sources), the cGMP/PKG pathway, and both CAMK II and p38MAPK (Fig. 3), all of which have been implicated in the diverse effects of CO, NO, or both gasotransmitters in other systems. Experiments reported in Fig. 6 indicate that the redox status of cysteine residues within Nav1.5 influence the channel sensitivity to CO. In line with a previous report in guinea pig myocytes (51), DTT was without significant effect itself on recombinant Nav1.5 at 1 mm (Fig. 7*A*). However, others have reported a modest augmenting effect of DTT on the cardiac Na^+^ channel (39) and a larger effect at higher concentrations in Nav1.5 derived from human jejunum smooth muscle (41). The most striking effect of DTT we found was to dramatically reduce the effect of subsequent exposure to CO, suggesting that CO sensitivity requires that key residues within the channel protein must be in an oxidized state. Yatsuhashi *et al.* (40) have reported that recombinant human cardiac Na^+^ channels expressed in COS7 cells are modestly inhibited by l-cysteine at the concentration reported here (100 μm; Fig. 7*C*). We did not detect such an effect, yet l-cysteine was not without influence since it significantly reduced the channel sensitivity to CO, as did the alkylating agent *N*-ethylmaleimide (Fig. 7*B*). Overall, these findings indicate that the redox state of key channel cysteine residues strongly influences the ability of CO to inhibit peak Na^+^ channel amplitude.

In summary, we have demonstrated that CO is a potent modulator of cardiac Na^+^ channels and that its ability to inhibit the peak Na^+^ current is mechanistically distinct from its ability to induce late Na^+^ currents. Although both effects involve formation of NO and are likely to be proarrhythmic, inhibition of peak Na^+^ currents cannot be mimicked by NO donors, do not appear to involve recognized signaling pathways, and are in part dependent on the redox status of the channel. Such actions of CO must be taken into consideration when evaluating this important gasotransmitter as a therapeutic agent.

## Footnotes

2 The abbreviations used are:

nNOS: neuronal nitric oxide synthase
DPBS: Dulbecco's phosphate buffered saline
MnTMPyP: Mn(III)terakis(1-methyl-4-pyridyl) porphyrin tetratosylate hydroxide
l-NAME: *N*^ω^-nitro-l-arginine methyl ester hydrochloride
eGFP: enhanced GFP
ROS: reactive oxygen species.
